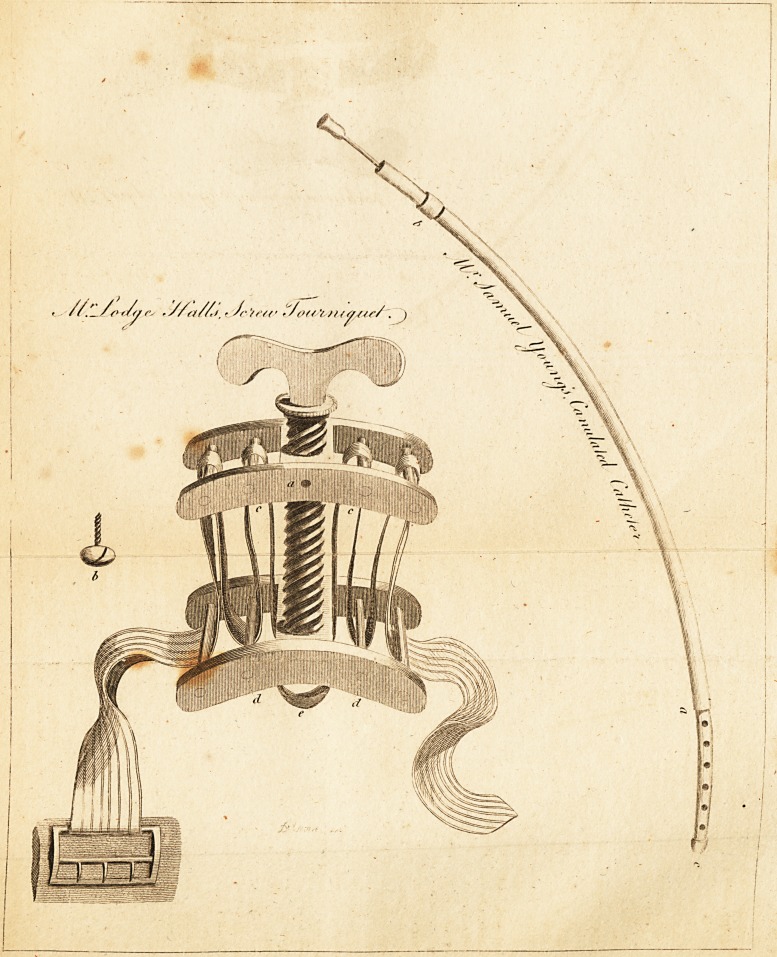# Mr. Young's Canulated Catheter

**Published:** 1805-12

**Authors:** Samuel Young

**Affiliations:** North Audley Street


### *To the Editors of the Medical and Phyſical Journal.*

Gentlemen,

I Beg leave to submit to your consideration the enclosed drawing of a Female Catheter, which Mr. Stothard has lately made from my instructions. *(See Engraving.)*

You will perceive that it consists of a catheter simply cased in a canula neatly fitted to it, but through which it slides with perfect freedom. For distinction's sake, I call it the CANULATED CATHETER. The canula is about the length of the common catheter; the catheter about two inches longer than the canula.

When it is to be introduced, the perforated head of the catheter is drawn back into the canula, home to the bulb *c*. In this state, the instrument exhibits merely a smooth blunt probe, and consequently having, in that state, no exposed perforations, nor any possible point of resistance on its surface, enters the part with as much facility and ease as seems to be possible. Being inserted into the bladder, and immersed in its contents, the catheter then, and not until then, is pushed forward out of the canula into

Mr. Lodge Hall's Screw Tourniquet.

Mr. Samuel Young's Canulated Catheter into the body of the urine; the wire is withdrawn, and the water discharged in the usual manner.

The drawing represents the canulated catheter in its parts, and the canula is described from *a.* to *b.* The structure of the instrument is so extremely simple, that I have not thought it necessary to add a distinct drawing of it as shut for introduction; trusting that it must be sufficiently obvious without it.

The chief OBJECT of this invention is to surmount a difficulty which, whenever it is present, must always render even so simple an operation as that of using the female catheter tedious and painful, if not sometimes wholly ineffectual. In using the *common* catheter, this difficulty must attend the process whenever the neck of the bladder is *diseased,* or even where there is any considerable increased action of the part for the time. A slight hæmorrhagy, in such instances, must commonly be one consequence, and the catheter will be choaked with coagulum, not unfrequently taking a complete cast of the whole tube. No stress need be laid on so obvious an inconveniency. In such circumstances, if the patient be relieved at all, the operation at best will be tedious and aggravating. These objections to the common catheter, as far as I perceive, will be completely obviated by the one I have now the honor to propose; in which the catheter itself is not exposed in the introduction, nor until the canula has passed the neck of the bladder, and is become stationary in the body of the urine. It can never therefore be clogged with coagulum, because the perforations, being concealed in the canula until the instrument is completely lodged within the bladder, will never be open for any blood to trickle in.

Another impediment that this canulated catheter would seem adapted to obviate, is that which arises from a severe spasmodic constriction of the neck of the bladder and urethra, and where the *exposed* perforations of the common catheter are grasped so firmly as to prevent its fair introduction. In an instrument constructed according to the suggestions I have offered, be the constriction what it may, it is the smooth surface of the *canula* only that will be grasped. The *catheter* is slipped into the bladder under the concealment of that surface, and when its office is performed, is returned by the same vehicle. I experienced lately the difficulties of both spasm and hæmorrhagy in a case of suppression, where Dr. Fraser attended. The patient had been subject to similar attacks, and the neck of the bladder der was considerably thickened. Upon passing the catheter, it was immediately filled by a cast of coagulum nearly through its whole length; and the catheter was so firmly grasped as to impede its fair introduction. The difficulties of this case first suggested to me the utility of having the holes of the catheter covered until they had fairly entered the bladder; and I also felt the necessity of not having the catheter exposed to the chance of being impeded by spasms; for in this instance had the instrument been cased, it might have been slipt into the centre of the bladder without the smallest difficulty.

I therefore trust, Gentlemen, that since the improvement I now submit to you, has been the direct invention of necessity, and is not one of those which require some invention to show their necessity, my wish for it to be known through so respectable a medium as the Medical and Physical Journal, will not be deemed impertinent.

I am, &c.

SAMUEL YOUNG.

*North Audley Street, Oct.* 1805.

## Figures and Tables

**Figure f1:**